# An Improved Pedestrian Ttracking Method Based on Wi-Fi Fingerprinting and Pedestrian Dead Reckoning

**DOI:** 10.3390/s20030853

**Published:** 2020-02-05

**Authors:** Bo Feng, Wei Tang, Guofa Guo, Xiaohong Jia

**Affiliations:** School of Electriacl and Control Engineering, Shaanxi University of Science and Technology, Xi’an 710021, China; wtang906@163.com (W.T.); guogf@126.com (G.G.); jiaxhsust@163.com (X.J.)

**Keywords:** Wi-Fi based fingerprinting, concentrated cost function, graph optimization, assisted positioning

## Abstract

Wi-Fi based positioning has great potential for use in indoor environments because Wi-Fi signals are near-ubiquitous in many indoor environments. With a Reference Fingerprint Map (RFM), fingerprint matching can be adopted for positioning. Much assisting information can be adopted for increasing the accuracy of Wi-Fi based positioning. One of the most adopted pieces of assisting information is the Pedestrian Dead Reckoning (PDR) information derived from inertial measurements. This is widely adopted because the inertial measurements can be acquired through a Commercial Off The Shelf (COTS) smartphone. To integrate the information of Wi-Fi fingerprinting and PDR information, many methods have adopted filters, such as Kalman filters and particle filters. A new methodology for integration of Wi-Fi fingerprinting and PDR is proposed using graph optimization in this paper. For the Wi-Fi based fingerprinting part, our method adopts the state-of-art hierarchical structure and the Penalized Logarithmic Gaussian Distance (PLGD) metric. In the integration part, a simple extended Kalman filter (EKF) is first used for integration of Wi-Fi fingerprinting and PDR results. Then, the tracking results are adopted as initial values for the optimization block, where Wi-Fi fingerprinting and PDR results are adopted to form an concentrated cost function (CCF). The CCF can be minimized with the aim of finding the optimal poses of the user with better tracking results. With both real-scenario experiments and simulations, we show that the proposed method performs better than classical Kalman filter based and particle filter based methods with both less average and maximum positioning error. Additionally, the proposed method is more robust to outliers in both Wi-Fi based and PDR based results, which is commonly seen in practical situations.

## 1. Introduction

While the problem of outdoor positioning has been extensively solved due to the developments of the Global Navigation Satellite System (GNSS), the indoor localization problem is still waiting for a satisfactory and reliable solution. Currently, many techniques have been applied to the area of indoor positioning. According to their different degrees of dependence on external infrastructures, these techniques can roughly be categorized into three types:

- Fully independent of external infrastructures. This type of positioning method relies on the inertial readings from waist mounted [1,2] or foot mounted [3,4,5] Inertial Measurement Units (IMUs) to calculate the relative position changes of the pedestrian. As the self-contained IMUs are adopted, no external signals or infrastructures are needed. Although the Pedestrian Dead Reckoning (PDR) algorithm [6] (corresponds to the waisted mounted situation) or the Zero Velocity Update (ZVU) algorithm [7] (corresponds to the foot mounted situation) can reduce the positioning error growth from cubic to linear, this type of method still has accumulative errors without upper bounds, without, however, the need for preinstalled devices. This type of method is thus suitable for emergency response cases, such as fire fighting.
- Fully dependent on special external infrastructures. This type of method or system relies on specially designed and deployed hardware (such as beacons or tags) in the positioning areas to provide the positioning signals. For example, the active bat system [8] and the Cricket system [9] adopt the deployed ultrasonic signals for positioning. The system SpotON [10] adopts the Radio Frequency Identification (RFID) technique to locate the pedestrian. Some other positioning systems, such as [11,12], adopt the Ultra Wide Band (UWB) system to locate the pedestrian according to Time of Arrival (ToA) measurements provided by the UWB systems. As this type of positioning system needs to deploy sensors or beacons all over the positioning areas, it is normally high cost and with limited coverage.
- Dependent on the near-ubiquitous signals in indoor environments. An example of this type is Wi-Fi based indoor positioning. As the Wi-Fi signals are already abundant in a wide range of buildings nowadays, they can be adopted without considering peculiar deployment. In this way, the cost can be lowered and the coverage significantly expanded.

In Wi-Fi based positioning, the user carries a Wi-Fi signal receiver—in most scenarios, a smartphone—-and needs to be located. From the receiver, Received Signal Strength Indication (RSSI) readings can be acquired for positioning use. The methods for Wi-Fi based positioning methods can roughly be categorized into two types: model based [13,14,15] and fingerprinting based [16,17,18]. Model based methods assume a path loss model to estimate the distances from the Access Points (APs) to the user. With the derived ranges, the position of the user can be estimated. However, as both the accuracy of the path loss model and the positions of APs are not always perfectly known, the overall accuracy of such a system can be greatly deteriorated. Fingerprinting based methods are arguably more accurate and more suitable for large scale use cases. Fingerprints in this context denote the RSSI readings from different APs and are considered unique, indicating different positions. The receiver can tell the RSSI readings from different APs because the APs are constantly sending messages containing information of their own Media Access Control (MAC) addresses. In this type of method, two steps are essential: the offline training phase and the online localization phase. In the offline training phase, a Reference Fingerprint Map (RFM) is established with fingerprints collected at different known locations called Reference Points (RPs). Then, in the localization step, a recently collected fingerprint is compared against the fingerprints in the RFM to solve for the position where the fingerprint is collected.

Much research has been done on the localization step. The RADAR system [19] proposed a simple k Nearest Neighbor (kNN) method for matching the RSSI fingerprints and solving for the location. Bayes’ rule is implemented in [20] and the localization problem is regarded as a Maximum A Posterior (MAP) estimation problem. However, these methods are computationally expensive with large Regions of Interest (RoI). To solve the problem of positioning latency due to larger RoI and to enhance accuracy, hierarchical positioning methods are proposed in [21,22]. In these methods, the RFMs are clustered into smaller batches or subregions either in the RSSI space or the coordinate space. The position of the user is first confined in one or several batches (coarse localization). Then comes the fine localization. As coarse localization can limit the search space to smaller regions, the positioning efficiency is increased. As the only hardware on the user side is normally a smartphone, other sensors mounted on the phone, e.g., inertial sensors, can be adopted to get superior positioning accuracy than pure Wi-Fi fingerprinting based positioning. Additionally, some other available information, e.g., indoor floorplans, can also be adopted. Generally, to include multiple sources of information to enhance Wi-Fi based fingerprinting is called assisted positioning. The authors in [23] adopted a particle filter (PF) to fuse the information of Wi-Fi fingerprinting positioning results, Pedestrian Dead Reckoning (PDR) information estimated from inertial sensors and indoor floorplans. The methodology proposed to form a discretized representation of the indoor map and then adopt it as a priori information in the PF probabilistic model. As some irrelevant degrees of freedom in the state space are removed, the methods can sufficiently decrease the number of particles needed while boosting accuracy. In [24], the authors proposed a particle filter method to integrate the fingerprinting and step counter results. This method is calibration-free for the step counter model and works transparently for heterogeneous devices and users. In [25], the authors combined the notions of hierarchical positioning and assisted positioning by designing a two-filter approach. The final positioning results are adopted for constraining the search space for fingerprinting matching. The first stage filter is a Kalman filter (KF) to achieve a smoothed fingerprinting matching. The second stage KF is adopted to integration of fingerprint matching results and PDR results. In [26], a PF was also adopted. The two primary sources needed to be integrated in the method are the Wi-Fi RSSI and PDR results. However, iBeacons are also deployed in areas with limited AP coverage and can be adopted to occasionally correct positioning errors. In [27], the authors again adopted a particle filter to integrate the information of Wi-Fi fingerprinting, PDR results and an indoor map. Differently, both fixed lag and fixed interval PF based smoothing processes were studied in the implementation, both of which can enhance accuracy and reliability compared to a conventional particle filter.

In the context of indoor pedestrian tracking from Wi-Fi fingerprint matching and other sources of information, most methods rely on filters such as KF and PF. Although some publications have made some modifications on the standard filter processes, e.g., by adding smoothing [27] and by adding multi-stage filter integration [25], they are still variants of filter based approaches. For these methods, no matter how the filters are implemented, e.g., Kalman filter or particle filter, they are assumed to be first-order Markov processes, denoting that the next estimation is only based on the previous estimation. All the previous information or observations are represented as the covariance matrix in the Kalman filter and a group of particles in the particle filter. In the structure of filter based methods, as the filtering process goes on, previous information is gradually “forgotten.”

**Figure 1:**
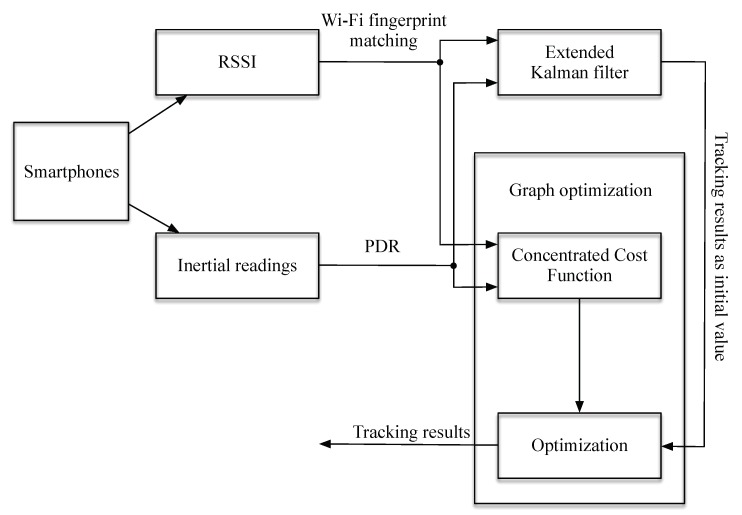
The framework of the proposed method for pedestrian tracking adopting Wi-Fi Received Signal Strength Indication (RSSI) and inertial measurements from a smartphone.

In this paper, a novel methodology for integration of Wi-Fi fingerprinting and PDR results is proposed. The methodology is graph optimization based rather than filter based. In the proposed graph-optimization based approach, all available observations, no matter whether they are old or current, contribute the same to the final concentrated cost function (CCF) and thus enable reaching globally optimized parameters, rather than sub-optimal estimations in filter based methods. The method only uses sensors from a Commercial Off The Shelf (COTS) smartphone. The framework of the methodology is shown in Figure 1, consisting of two blocks. The filter block is essentially a simple extended Kalman filter (EKF) integrating Wi-Fi fingerprinting and PDR results for tracking. The tracking results are adopted as initial values for the optimization block, while Wi-Fi fingerprinting and PDR results are adopted to form an CCF. The CCF can be minimized with the aim of finding the optimal poses of the user with better tracking results. As the EKF tracking results can normally provide sufficiently good initial values, the optimization can reach convergence in only a few iterations. This means that the optimization has the ability to run on real time. Experiments were designed to verify that the proposed methodology can outperform classical KF and PF based methods in terms of pedestrian tracking accuracy. Simulations were carried out, showing that the proposed methodology is more robust to noises or errors in both Wi-Fi fingerprint matching results and PDR results. The fingerprinting errors are by far the most prevalent in practical situations due to many reasons, such as constant changes in the RFM and RSSI measurement noises. PDR errors are also very common in real applications, possibly due to unconventional movement of the smartphone carriers.

The remainder of the paper is arranged as follows: Section 2 gives the related works on pedestrian tracking, including the PDR algorithm and graph optimization, which are used as tools for solving the pedestrian tracking problem in this paper. Section 3 is the methods section, describing the details of the proposed method (Figure 1). Section 4 is the experiment section with both simulations and real-scenario experiments. Then comes the conclusion (Section 5) and the discussion (Section 6).

## 2. Related Works

The PDR algorithm has become more and more common in many smartphone based applications because it is an efficient way for tracking the user based on inertial readings [28,29]. Graph optimization, as an alternative to filter based methods, has gained more and more attention over the years, and has been proven to have more accurate results than filter based methods in vision based positioning [30,31,32]. The PDR algorithm and graph based optimization are adopted as two tools for solving the pedestrian tracking problem in this paper. They are introduced as follows.

### 2.1. Pedestrian Dead Reckoning

Unlike closed form inertial calculations, the PDR algorithm for pedestrian tracking assumes a step wise motion model of the user; i.e., the user walks step by step. This can be described in Equation (1):

(1) $$\begin{array}{c} x_{t}=x_{t-1}+L_{t}cos\left(\varphi_{t}\right)\\ y_{t}=y_{t-1}+L_{t}sin\left(\varphi_{t}\right) \end{array}$$

where $x_{t}$ and $y_{t}$ denote the user’s position of the $t^{th}$ step. The position can be derived from the previous step if the step length $L_{t}$ and the heading $\varphi_{t}$ are known. In order to track the user’s positions, the PDR algorithm should provide the three types of corresponding information in the following ways:

- Detecting new steps. This involves activity recognition based on inertial readings. The user can carry the smartphone while at standstill, moving forward and during other irregular movements. This issue has been studied in publications such as [28]. If new steps were detected falsely, the tracking error will increase.
- Step length estimation. This corresponds to the estimation of $L_{t}$ from Equation (1). This can not be done through inertial calculation because there is too much noise in the inertial readings and the error accumulates cubically. In our paper, we adopt the a priori model, which assumes that the step length is linear to the step frequency.
  (2) $$L_{t}=af_{t}+b$$
  where $f_{t}$ is the frequency of new steps and parameters *a* and *b* are related to the users. From publication [33], these parameters can be adaptively estimated during walking. According to the method, minimum training data are needed for the relationship between *a*, *b*, step frequency and step length for different individuals. Therefore, there is a need for minimum "offline" training phase for the step length estimation.
- Orientation or heading estimation. This corresponds to the estimation of $\varphi_{t}$ from Equation (1). In this paper, we adopt the method proposed in [34], where the magnetometer readings can be adopted to improve heading estimation. The method in [34] also has the advantages of low computational load and is able to operate under low sensor sampling rates. This is in favor of limiting the needed computing power and enabling real time implementation.

Although many methods have been proposed to enhance the accuracy of the PDR algorithm, tracking results based on pure PDR are still quite unreliable due to the fact that the inertial sensors on a smartphone are low end inertial sensors. In general, the tracking results from the PDR algorithm are integrated with other types of information to work. In our paper, the other type of information is the Wi-Fi based fingerprint matching.

### 2.2. Graph Optimization

Graph optimization can be regarded as the counterpart of recursive filters. In recursive filters, such as in KF or PF, the newest state or estimation is made with the latest observation and the previous state estimations. While in graph optimization, the states in different times are estimated in a batch by minimizing the CCF composed of square error terms between the observations and the states at different times. It is a state-of-the-art technique widely adopted in the visual based positioning area, especially for the visual based Simultaneously Localization and Mapping (SLAM) problems.

In the graph optimization problem, the CCF can be represented as square sums, and can be related to a graph, where the nodes can represent the states or variables to be estimated and the edges between the nodes can present a known relationship between the nodes. Minimizing the CCF is essentially finding the best configuration of nodes which satisfies the known relationships (i.e., observations) best [35]. As the CCF has the square sum terms, the optimization problem can be regarded as the minimization of non-linear least squares. The typical solution for such a problem is to linearize around the current state, solve a linear system and then iterate. For solving the linear system, typical methods include Gauss-Newton algorithm and Levenberg-Marquardt algorithm. The solution for the graph optimization problem was surveyed in detail in [36], and we directly took the functional tools from it.

In the context of pedestrian positioning, the tool of graph optimization mostly focuses on building the RFM. In [37], the authors adopted the tool for building the RFM with a foot-mounted Inertial Measurement Unit (IMU) and a smartphone. In [38], the tool was adopted to merge crowd-sourced trajectories and generate the RFM with the help of some known landmarks. In those methods, other external devices or information, e.g., Bluetooth beacons, external inertial measurements units (IMUs) or landmarks with known positions are needed, which increases the system complexity, hindering them from prevalent usage in indoor environments. The proposed method, however, focuses on the positioning part, and only relies on a smartphone on the user side, which is suitable for ubiquitous indoor positioning. In our paper, we focus on pedestrian tracking adopting the tool by integration of PDR results and Wi-Fi fingerprint matching. Here, we do not go to detail for the optimization process but rather focus on how to fit the mentioned pedestrian tracking problem with the graph optimization framework.

## 3. Method

As shown in Figure 1, two types of readings from the smartphones are adopted for positioning: RSSI values and inertial readings. The RSSI values are adopted for the Wi-Fi based fingerprint matching algorithm, which can estimate the locations of the users. More of the matching algorithm implemented here will be discussed in Section 3.1. The inertial readings are processed through the PDR algorithm. As mentioned in Section 2.1, the step detection and the step length were estimated adaptively from [33], and the orientation estimation was taken from the method in [34]. Because these are widely adopted, mature methods, we do not include the details in the scope of this paper. For the Wi-Fi based fingerprinting results, the position estimation may have some outliers due to the noise in both the RFM and the measured RSSI. For the PDR based tracking results, the positioning error may accumulate in time severely. These two estimations are considered independent because they come from different sensors on the phone. They also have different error features and can be integrated to achieve better results. The EKF (introduced in Section 3.2) is firstly carried out to integrate the Wi-Fi based fingerprinting results and the PDR results. A rough fusion output can then be acquired. The output acts as the initial value for the graph optimization block (including the establishing of the CCF and the process for optimization). The outputs of the graph optimization block are the final tracking results, which are provably more accurate than the output from the EKF. The graph optimization block is introduced in Section 3.3.

### 3.1. Wi-Fi Based Fingerprint Matching

**Figure 2:**
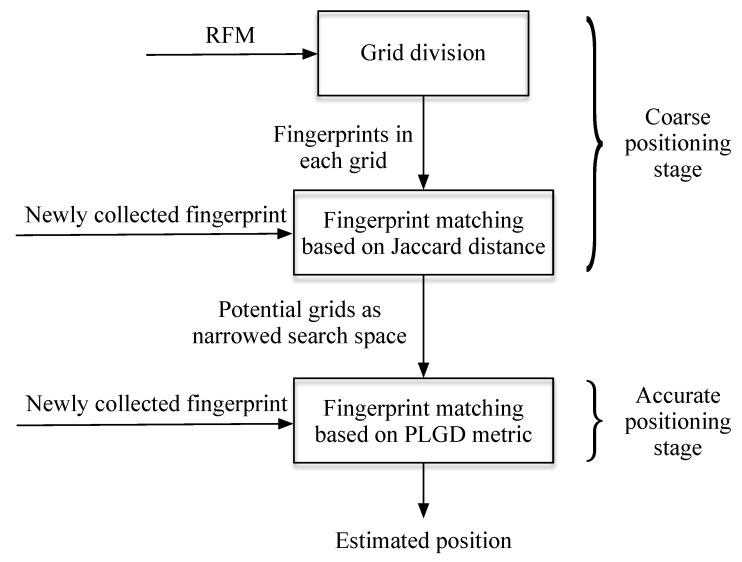
The hierarchical structure for Wi-Fi based positioning in our implementation.

The Wi-Fi based fingerprint matching algorithm is the key for Wi-Fi based positioning. When without any additional data to assist positioning, the matching results can be directly adopted as the location estimations of the users. In our implementation, we adopt the hierarchical structure for Wi-Fi based positioning. The hierarchical based positioning can significantly lower the computational cost if the RFM covers a large area [21]. The structure of the hierarchical based Wi-Fi fingerprint matching is shown in Figure 2. It has two stages: the coarse positioning stage and the accurate positioning stage. In the coarse stage, the positioning area is partitioned into larger grids. The representing fingerprint for the area is represented as the set as all the available APs in the grid:

(3) $$O_{i}=\cap_{F_{j}\in a_{i}}AP\left(F_{j}\right)$$

where $F_{j}$ is the fingerprint (vector of RSSIs from different APs), $a_{i}$ denotes the area of the grid *i* and the function AP(.) returns the set of available APs of the fingerprint. For a newly collected fingerprint $F_{new}$, the task is to compare it with all the representing fingerprints in the grid areas, and choose several of the closest grids as the potential area. During the comparison, we adopt a simple Jaccard distance:

(4) $$d_{coarse}\left(O_{new},O_{i}\right)=\frac{|O_{new}\cup O_{i}|}{|O_{new}\cap O_{i}|}$$

where $O_{new}$ denotes the AP set of the newly collected fingerprints $F_{new}$ and the operator |.| denotes the cardinality of the set. The Jaccard distance is adopted in a similar manner in [39]. The distance metric is generally faster because it does not need to complement the fingerprints to vectors of the same length. However, also because the Jaccard distance only takes the availability of APs into consideration, it is not as accurate as those metrics adopting the actual values of RSSIs as well. This metric is suitable for use in the coarse localization stage because we only need to narrow the potential area to a small number of grids.

As mentioned, in accurate localization phase, as the search space is narrowed, the matching process can be accelerated. Here we implement the Penalized Logarithmic Gaussian Distance (PLGD) distance as the metric between two fingerprints. The PLGD metric is proposed in [40] and has better performance than a traditional Logarithmic Gaussian Distance (LGD), which is defined as:

(5) $$d_{LGD}\left(F_{i},F_{j}\right)=-\sum_{k}\log max\left(G\left(F_{i,k},F_{j,k}\right),\epsilon \right)$$

The index *k* means the indexes of APs shared by $F_{i}$ and $F_{j}$, and the respective RSSI values are $F_{i,k}$ and $F_{j,k}$. The function G(.) can be written as:

(6) $$G\left(F_{i,k},F_{j,k}\right)=\frac{1}{\sqrt{2\pi \sigma^{2}}}\exp\left(-\frac{{\left(F_{i,k},F_{j,k}\right)}^{2}}{2\sigma^{2}}\right)$$

The max operation in Equation (5) gives an upper bound of ϵ if $G(F_{i,k},F_{j,k})$ is close to zero. However, the LGD metric does not consider the situation where some APs are available in $F_{i}$ but not in $F_{j}$ or vise versa. Therefore, PLGD adds the terms to give a penalty for the situation:

(7) $$d_{PLGD}\left(F_{i},F_{j}\right)=d_{LGD}\left(F_{i},F_{j}\right)+\alpha \left(\phi \left(F_{i},F_{j}\right)+\phi \left(F_{j},F_{i}\right)\right)$$

where the coefficient α is a hyperparameter indicating the weight of the penalized terms and is studied in the experimental section. The function ϕ(.) is

(8) $$\phi \left(F_{i},F_{j}\right)=\sum_{k}\left|\lambda -F_{i,k}\right|$$

where the index *k* represents the indexes of APs in $F_{i}$ but not in $F_{j}$, and λ is a default RSSI value indicating a missing measured attribute. In our implementation, this value was set to −100 dBm. The PLGD metric considers both the availability information and the RSSI values in the fingerprints, and was thus adopted for our method.

### 3.2. EKF for Integration

To integrate the information of PDR based estimations and Wi-Fi fingerprint matching based estimations, a EKF is adopted here. Normally, the EKF includes two phases: the prediction phase and the measurement correction phase. In our implementation, the prediction denotes adopting the step length and heading change from the PDR algorithm to make the prediction of the person’s position in the next step. The measurement correction phase denotes correct the errors in PDR based tracking according to the estimated positions from Wi-Fi based fingerprint matching.

The person’s state can be represented as the horizontal positions and the heading:

(9) $$s_{t}=\left[\begin{array}{c} x_{t}\\ y_{t}\\ \varphi_{t} \end{array}\right]$$

The prediction can be written as:

(10) $$s_{t+1}=s_{t}+\left[\begin{array}{c} L_{t}cos\left(\varphi_{t}+\delta \varphi_{t}\right)\\ L_{t}sin\left(\varphi_{t}+\delta \varphi_{t}\right)\\ \delta \varphi_{t} \end{array}\right]$$

Here $L_{t}$ and $\varphi_{t}$ are the estimated step length and the heading change from the PDR algorithm. Then the covariance of the state $P_{t}$ is updated as:

(11) $$P_{t+1}=A_{t}P_{t}A_{t}^{T}+W_{t}Q_{t}W_{t}^{T}$$

As Equation (10) means that the prediction is a none linear process, when solving for the covariance update, it should be linearized. Then, matrix $A_{t}$ is the Jacobian of Equation (10) over the state s. Similarly, matrix $W_{t}$ is the Jacobian matrix over the “driven vector” $\left[L_{t},\delta \varphi_{t}\right]$. In this implementation, $A_{t}$ can be written as:

(12) $$A_{t}=\left[\begin{array}{ccc} 1 & 0 & -L_{t}sin\left(\varphi_{t}+\delta \varphi_{t}\right)\\ 0 & 1 & L_{t}cos\left(\varphi_{t}+\delta \varphi_{t}\right)\\ 0 & 0 & 1 \end{array}\right]$$

and $W_{t}$ can be written as

(13) $$W_{t}=\left[\begin{array}{cc} cos(\varphi_{t}+\delta \varphi_{t}) & -L_{t}sin\left(\varphi_{t}+\delta \varphi_{t}\right)\\ sin(\varphi_{t}+\delta \varphi_{t}) & -L_{t}cos\left(\varphi_{t}+\delta \varphi_{t}\right)\\ 0 & 1 \end{array}\right]$$

$Q_{t}$ is the process noise, and here we assume that it is linearly related to the estimated step length and heading change form the PDR algorithm:

(14) $$Q_{t}=\left[\begin{array}{cc} aL_{t} & 0\\ 0 & b\delta \varphi_{t} \end{array}\right]$$

where a,b are the linear coefficients.

In the measurement correction phase, as two of the elements of the state $s_{t}$ are directly observable, the measurement model is deemed as linear. The observation matrix can be written as:

(15) $$H_{t}=\left[\begin{array}{ccc} 1 & 0 & 0\\ 0 & 1 & 0 \end{array}\right]$$

Then the measurement correction calculation can be written as

(16) $$\begin{array}{c} K_{t}=P_{t}H_{t}\left(H_{t}P_{t}H_{t}^{T}+R\right)\\ s_{t}=s_{t}+K_{t}\left(Z_{t}-H_{t}s_{t}\right)\\ P_{t}=\left(I-K_{t}H_{t}\right)P_{t} \end{array}$$

where

- R is the noise variance matrix for the Wi-Fi based position estimation;
- $Z_{t}$ is the position estimation from the Wi-Fi based fingerprint matching process;
- $K_{t}$ is the Kalman gain;
- In this process, the predicted s and P from the prediction step are corrected according to the observation $Z_{t}$.

There are some outliers from the Wi-Fi based fingerprint matching process. However, we do not take out the outliers in the EKF. The integration results from the EKF are only rough estimations and need to be further taken care of.

### 3.3. CCF Forming and Graph Optimization

**Figure 3:**
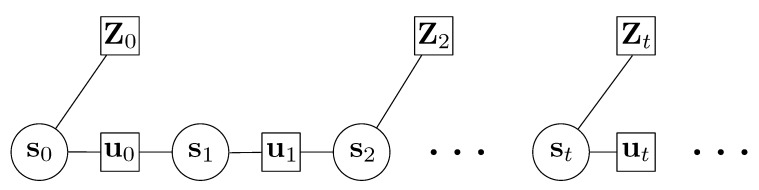
The graphical representation for the problem of integration of Pedestrian Dead Reckoning (PDR) based position estimations and Wi-Fi based estimations.

As mentioned, unlike recursive filters, the graph optimization can adopt all the available measurements to estimate the positions in a batch. The key for graph optimization is to construct a graph or CCF to minimize by varying the state estimations. In the problem of integration of PDR and Wi-Fi based fingerprinting, the overall CCF should include both sub-terms in the form of square errors. The CCF can also be represented as a graph shown in Figure 3. Each node (circle) denotes a state variable to be estimated, and here the nodes indicate the state or poses of the person. The squares denote the available measurements. There are two types of measurements here, and they are the pose changes (including step length and heading change) from the PDR algorithm and the position estimations from the Wi-Fi based fingerprint matching. The edges connecting the nodes mean that the corresponding observation can represent some relationship of the nodes. In other words, a cost term can be formed between the observation and corresponding nodes. The symbols in the graph representation are:

- $s_{t}$ are the poses of the person to be estimated;
- $u_{t}$ denotes the estimations from the PDR algorithm and $u_{t}={\left[L_{t},\delta \varphi_{t}\right]}^{T}$;
- $Z_{t}$ means the two-dimensional position estimations from Wi-Fi based positioning and can be written as $Z_{t}={\left[x_{z,t},y_{z,t}\right]}^{T}$.

As can be seen in Figure 3, there are two types of edges; correspondingly, there are also two types of error terms in the CCF. We firstly form the PDR based error terms. From the variables from $s_{t}$, we can derive the pose change $u_{t}^{s}$ between the previous time and the current time:

(17) $$u_{t}^{s}=\left[\begin{array}{c} \sqrt{{\left(x_{t}^{s}-x_{t-1}^{s}\right)}^{2}+{\left(y_{t}^{s}-y_{t-1}^{s}\right)}^{2}}\\ \varphi_{t}^{s}-\varphi_{t-1}^{s} \end{array}\right]=\left[\begin{array}{c} L_{t}^{s}\\ \delta \varphi_{t}^{s} \end{array}\right]$$

where $\left[x_{t}^{s},y_{t}^{s},\varphi_{t}^{s}\right]$ are taken from the variables of $s_{t}$. Then we can form the cost (in a square sum form) denoting the differences between the pose changes derived from PDR and pose changes derived from the state variables as:

(18) $$C_{PDR}=\sum_{k}e_{k}^{PDR}W^{PDR}{e_{k}^{PDR}}^{T}$$

where *k* denotes indexes on all steps, $W^{PDR}$ denotes a 2×2 weight matrix for the cost and $e_{k}^{PDR}$ is

(19) $$e_{k}^{PDR}={\left[\begin{array}{c} L_{t}-L_{t}^{s}\\ \delta \varphi_{t}-\delta \varphi_{t}^{s} \end{array}\right]}^{T}$$

which is composed of the step length and heading differences. If the state variables are exactly the same with the PDR derived results, $C_{PDR}$ should be 0.

The costs derived from Wi-Fi based fingerprint matching are similar. They represent the differences between the state variables and the estimations from Wi-Fi positioning. The cost can be written as:

(20) $$C_{WiFi}=\sum_{q}e_{q}^{WiFi}W^{WiFi}{e_{q}^{WiFi}}^{T}$$

where *q* is the indexes over all WiFi based position estimations; $W^{WiFi}$ is the weight matrix, also being 2×2 dimensional; and $e_{q}^{WiFi}$ is

(21) $$e_{q}^{WiFi}=Z_{q}-\left[\begin{array}{c} x_{q}^{s}\\ y_{q}^{s} \end{array}\right]$$

$Z_{q}$ means the two-dimensional position estimations from Wi-Fi fingerprinting. Similarly, if the states’ variables are the same as the Wi-Fi based results, the Wi-Fi cost term should be 0.

For the weights of WiFi based cost function, as there should be no differences in error contributions between the errors in x-axis and y-axis, we define each as:

(22) $$W^{WiFi}=\left[\begin{array}{c} 1,0\\ 0,1 \end{array}\right]$$

For the PDR based cost function, we define the weight matrix as follows according to the contribution in [41]:

(23) $$W^{PDR}=\left[\begin{array}{c} 1,0\\ 0,10 \end{array}\right]$$

which implies that the heading error contributes 10 times more than the step size error. As the weights for the PDR cost function were already studied in publication [41], here we directly take the value.

Then, the CCF is the sum of the PDR terms and Wi-Fi terms:

(24) $$CCF=aC_{PDR}+\left(1-a\right)C_{WiFi}$$

**Figure 4:**
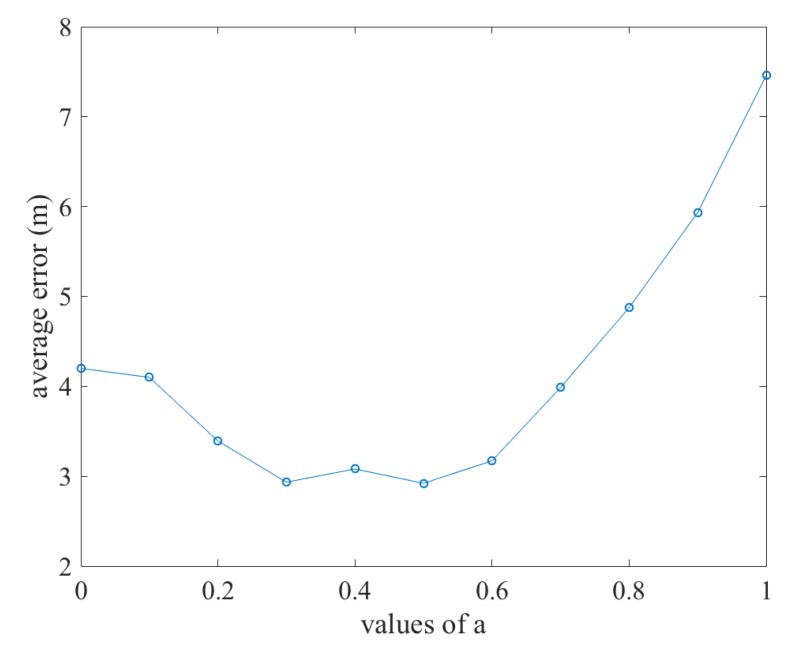
Average positioning error over different values of hyper-parameter *a*.

For the weights of the CCF terms, we can assume that the weights are summed to be 1. This assumption is reasonable because any scale factor of the weights would not affect the results of the minimization of the CCF. The symbol *a* is a hyper-parameter defining the weights of the CCF terms. The value of *a* should be between 0 and 1. If it is 0, then the fusion result would degenerate to pure Wi-Fi fingerprinting based results, and otherwise to pure PDR results. In our method, we vary the value of *a* from 0 to 1 spaced at 0.1. Figure 4 shows the average positioning errors with different values of *a*. We can see that the curve is flat in the center and peaks at the two ends. The peaks denote that only one type of information between Wi-Fi fingerprinting and PDR results is adopted. We can also see from the figure that the flat area ranges from about 0.3 to about 0.6. In this range, the average positioning error only has minor fluctuations. This means that the average positioning error is not sensitive to the value during this range. In our implementation, we adopted the value 0.4. To sum it up, here only one of the weights factors was considered as a hyper-parameter to tune prior to the implementation to the approach. Other weights were directly taken from previous publications. It was just a current implementation, and how the overall weights will affect our method will be studied in the future.

The minimization problem for the CCF is a classical square sum minimization problem, and has been solved with some compact tools like G2O [36]. Another thing to be noted is that there are some outliers in the measurements, including PDR results due to false step detection and Wi-Fi positioning results due to changes in the RFM and noises in the collected RSSI fingerprints. To overcome the outlier problem, we have added a Huber loss function on the CCF

(25) $$CCF=\sum_{n}\rho \left(r_{n}\left(x\right)\right)$$

where ρ(.) is the Huber loss function, *n* represents the indexes over all the square terms, $r_{n}\left(\right)$ is the residual error and x denotes all the variables to be estimated. The Huber loss function is added to avoid the phenomenon that outliers can introduce large residuals. The adding of loss functions on the CCF is a technique called iterative least-squares, and is introduced in detail in [36]. The complexity for the optimization is $O(N^{2})$, where N is the dimension of the state space, herein the number of poses to be estimated.

## 4. Experiment

Both a real-scenario experiments and simulations were carried out to verify the effectiveness of the proposed method. Since Wi-Fi based fingerprint matching can be considered as a fundamental for the proposed methodology, it was tested, at first showing that the implementation here (specifically the hierarchical structure and the PLGD metric) is effective at increasing the positioning accuracy solely from Wi-Fi fingerprints. Then, the performance of the proposed integrated positioning methodology adopting PDR and Wi-Fi fingerprint matching was tested and compared with traditional integration methods such as EKF and particle filtering. Besides experiments adopting data collected in real-scenarios, we also carried out simulations by manually adding outliers in positioning results from Wi-Fi fingerprint matching and PDR. Then we tested the robustness of the proposed graph optimization based methodology. Note that the positioning outliers for Wi-Fi based fingerprinting and PDR result matching can be very common in practical situations.

### 4.1. Experimental Settings

To test the performance of the positioning methodology, two fundamental issues should be considered.

- The prerequisites are needed for Wi-Fi based fingerprinting matching. Most importantly, the RFM should be established in the experimental scene.
- The positioning benchmarks should be established. This raises the issue of how to acquire the ground truth positions to get the positioning errors and the statistics of the positioning errors.

**Figure 5:**
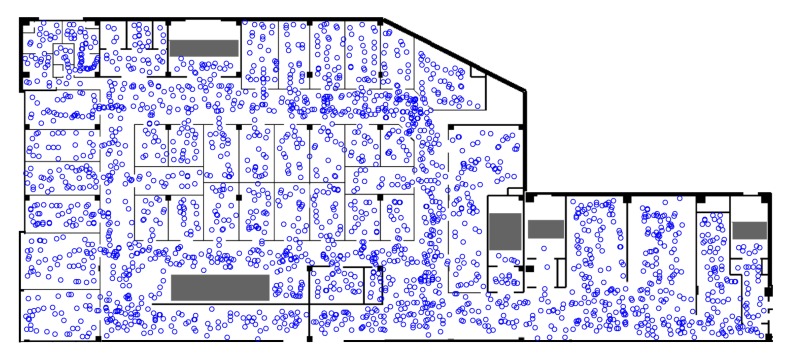
The experimental scene at a mall along with the points where fingerprints were collected.

We designed the experiments considering the aforementioned fundamental issues. The experiments were carried out in a partial part of a mall, as shown in Figure 5. There were already abundant APs installed in the mall, so there was no need for further installation of APs for the experiments. To solve for the first issue, we adopted the method in [40] for generating the RFM. Specifically, we acquired the map of the mall, as shown in Figure 5. The Nexus 6P device (Android OS) was adopted to collect the Wi-Fi signal fingerprints. The person carrying the device was asked to walk around in the mall and to mark positions by clicking on the device’s screen. Then, according to the marking time of the positions and the Wi-Fi fingerprinting collecting time, the locations of the reference points were interpolated. The RFM was established according to the fingerprints and the interpolated positions. For the second issue, the ground truth positions were measured in a similar way by interpolation according to the marking time and the fingerprints collecting time. In Figure 5, it can be seen that the positions are spaced in nearly all the floorplan. In our experiment, the total number of points was 2373. This number includes the reference points (70%) adopted for establishing the RFM and the ground truth positions (30%) for testing accuracy. The locations of the points look a bit random for the following reasons: (1) the sampling interval of the Wi-Fi fingerprints is not a constant in the actual data collection process due to the scanning mechanism; (2) the walking trajectories of the person have some randomness. The aforementioned scheme for labeling coordinate positions is not perfect. The coordinate position accuracy highly depends on each data collector’s marking accuracy. However, this method is widely adopted because the effort needed for labeling is much less than site surveying using a total station.

For the purpose of data collection, an Android application was developed. The Android application can:

- Show the map of the mall;
- Log the marking time and positions;
- Log the collected Wi-Fi fingerprints and the collecting time;
- Log the inertial measurements with time.

Most importantly, the different types of data have synchronized timestamps, and thus can be adopted for interpolation as aforementioned. Another thing should be noted is that the positioning errors statistics were all based on the positions at the fingerprinting collecting time, not the times of steps (PDR epochs). Even though the position integration returned the positioning estimations at each step, we adopted the estimations to derive the position estimations at Wi-Fi fingerprint collecting time to show the error statistics.

### 4.2. Wi-Fi Based Fingerprint Matching

**Figure 6:**
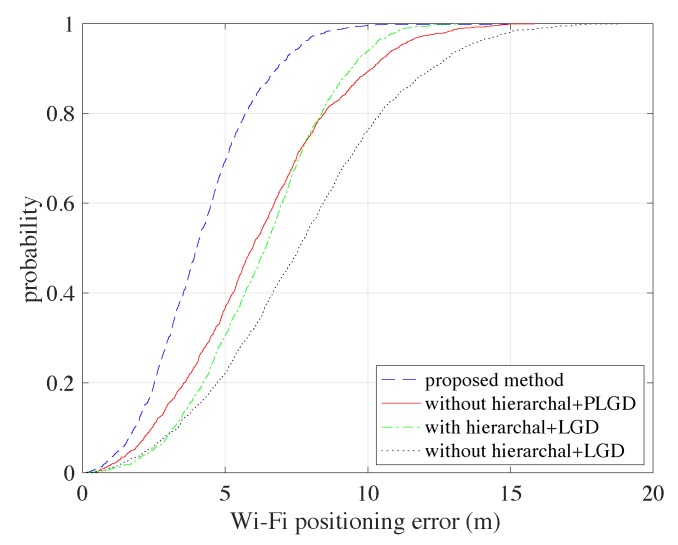
Wi-Fi fingerprinting based positioning results comparisons using different strategies.

The Wi-Fi fingerprint matching in our methodology implements a hierarchical structure and the PLGD metric. Wi-Fi fingerprint matching can be considered as a fundamental work for our methodology, which can provide the Wi-Fi based positioning estimations for integration. Here we carried out experiments showing that the proposed fingerprint matching method has better performance by adopting the hierarchical structure and the PLGD metric. Figure 6 shows the Cumulative Density Functions (CDFs) comparisons of the different combinations: with hierarchical+PLGD, without hierarchical+PLGD, with hierarchical+LGD, and without hierarchical+LGD. We can see that adopting both the hierarchical structure and the PLGD metric can increase the positioning performance. Specifically, applying the hierarchical structure can bring an average error decrease of about 2.0 m. By applying PLGD the number is about 2.1 m, and by applying both the number is 3.5 m.

**Figure 7:**
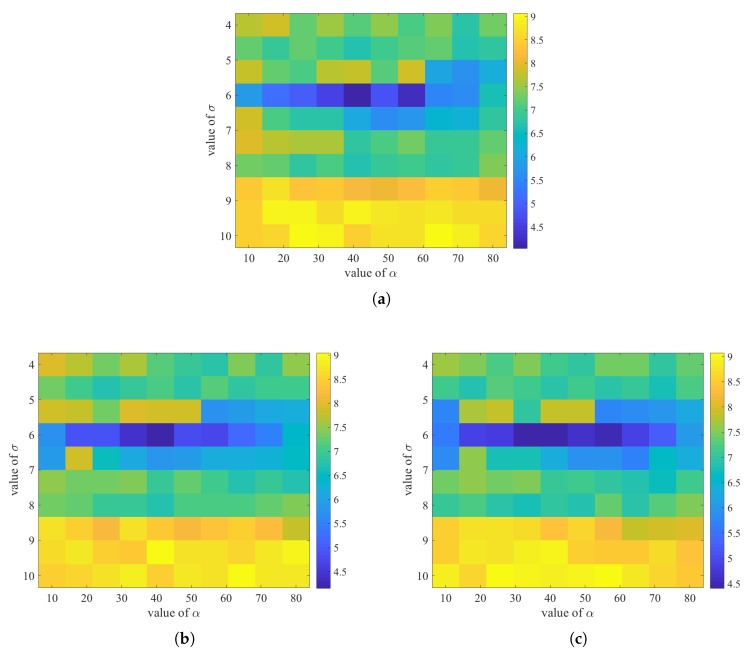
Two-dimensional grid search for the hyperparameters α and σ for Wi-Fi based fingerprinting if (**a**) grid length is 1 m; (**b**) grid length is 1.5 m; (**c**) grid length is 2 m.

In the proposed Wi-Fi based fingerprinting, some hyper-parameters have effects over the positioning accuracy. The hyper-parameters include the grid length, σ and α (adopted in metric definition in the fingerprinting space). To define the best hyper-parameters, a three-dimensional search is normally needed. However, instead of performing the three-dimensional optimal hyper-parameter search, we only performed a two-dimensional search (over σ and α ) assuming typical grid length values (1, 1.5 and 2 m). This is because that dividing the coordinate space into grids is not new in Wi-Fi based positioning. We found out that the grids were adopted in publications such as [21,39,42]. Typical grid length ranges from 1 to 2 m in the mentioned papers. The two-dimensional search results are shown in Figure 7; we found out that the average positioning errors do not change much if the grid length values are within the three typical values. This might be the reason why many publications do not list the value of grid length as one of the hyper-parameters to tune prior to positioning. In our method, we adopted 1.5 m as the grid length value. The corresponding two-dimensional hyper-parameter search results when the grid length equals 1.5 are shown in Figure 7b. Recall that σ and α denotes the variance of the LGD and the penalty coefficient. The ranges of the σ and α are limited according to their typical values from other implementations [40]. The search resolution of σ is about 0.67 and that of α is about 7.8. From Figure 7b, we can see that the best hyper-parameters herein for σ and α are 6.7 and 33.3 respectively.

### 4.3. Integration of PDR and Wi-Fi Based Fingerprinting

By walking in the experimental scene and marking on the map, we can establish a RFM, as mentioned before. Then we can collect some test data which includes Wi-Fi fingerprints, inertial measurements and marking positions. With these data, we can achieve the ground truth positions of the person (through linear interpolation according to time) and can test the performance of the proposed methodology. Both a real-scenario experiment and simulations were carried out for such purposes.

#### 4.3.1. Real-Scenario Data Experiment

**Figure 8:**
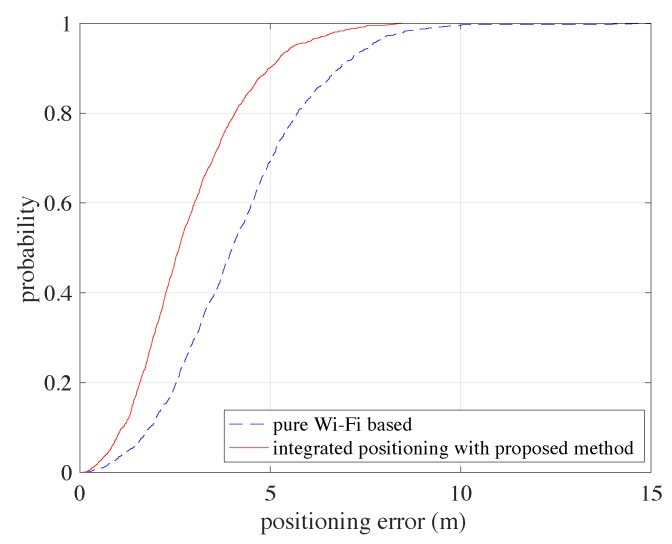
The Cumulative Density Function (CDFs) comparisons between pure Wi-Fi based positioning and the integrated positioning using the proposed methodology.

Figure 8 shows the positioning error CDF comparisons of the proposed methodology and the pure Wi-Fi based positioning without the assisting of PDR information. It shows that the PDR information is very useful for helping increasing the positioning performance.

**Figure 9:**
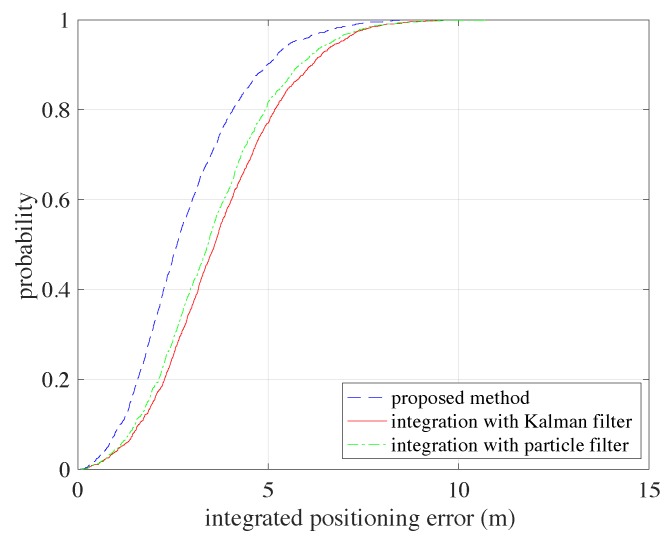
The CDF comparisons between the proposed methodology, the Kalman filter based methodology and the particle filter based methodology.

Figure 9 shows the positioning error CDF comparisons between the proposed graph optimization methodology, Kalman filter based integration method (the results are taken from the descriptions in Section 3.2) and particle filter based integration method. Note that the Kalman filter based integration can provide the initial values for graph optimization in the proposed methodology. Not surprisingly, our method provides the best positioning performance, with a CDF above the other’s CDF values. This is because our method can be regarded as having adopted all the available information for estimation, while the Kalman based method and the particle filter based method can only adopt the previous measurements for position estimation at current time.

The results from both Figure 8 and Figure 9 are summarized in Table 1. We can see that integration of PDR information can indeed increase positioning performance regardless of which integration method is adopted. The average positioning error of the proposed method is 0.8 m and 0.7 m less than Kalman filter based method and particle filter based method respectively. Additionally, using the proposed method, the maximum positioning error is greatly decreased, by 1.3 m compared with the Kalman filter based method and by 1 m from the particle filter based method. The dropping of maximum positioning error can show that the proposed method is more robust than the other two integration methods.

#### 4.3.2. Simulations

In practical Wi-Fi fingerprinting and PDR based positioning, there may be several outliers due to a large variety of reasons. For Wi-Fi based fingerprinting, the positioning results from Wi-Fi based fingerprint matching are often not as good as in Section 4.2. This is because that the RFM is constantly changing in the environment. There are many factors causing such changes, including the moving object and people, the installations of APs, the moving of existing APs and so on. These changing factors can cause the positioning errors from Wi-Fi based fingerprint matching to increase; in particular, they can bring outliers into position estimations. For PDR based positioning, the outliers may originate from bad accuracy in the devices’ inertial sensors and irregular walking patterns or device placement patterns. These factors can be commonly seen in practical applications and need to be taken into consideration. Therefore, to dissect into the robustness of the proposed method, we performed simulations which introduced different levels of outliers both in Wi-Fi fingerprinting and PDR positioning. Specifically, for Wi-Fi positioning, we manually added Wi-Fi positioning outliers to 5% and 10% of the data. Table 2 and Table 3 show the positioning results comparisons of the three integration methods. Here, we added the outliers by randomly choosing a specific percentage of Wi-Fi based positioning results and adding errors uniformly distributed at a range between 10 and 20 m. For PDR based positioning, outliers were added by introduce larger errors in pose changes. For that, we also took 5% and 10% of all PDR based pose changes as outliers. In these outliers, we introduced a uniformly distributed step length scale factor between 0.5 to 2, and a heading change scale factor, also from 0.5 to 2. Table 4 and Table 5 show the respective error comparisons of the proposed method, traditional Kalman filter based method and particle filter based method.

**Figure 10:**
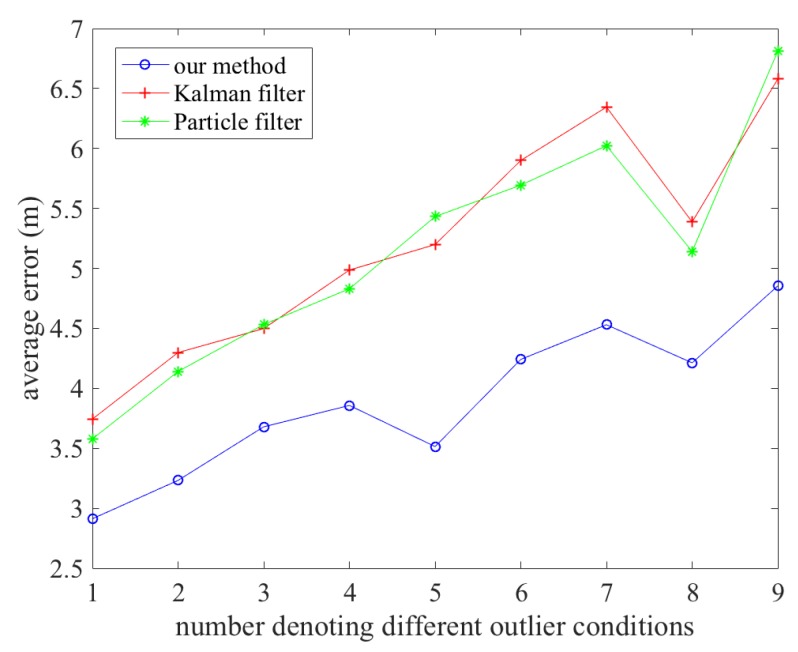
Average positioning comparisons of our method, the Kalman filter based method and the particle filter based method over different outlier conditions.

Figure 10 shows the average error curve comparisons of the three methods in different outlier conditions. The horizontal axis denotes the different outlier conditions and the vertical axis denotes the average positioning errors. Table 6 shows the how the outlier conditions correspond to the numbers in the horizontal axis. From this figure, we can see that under all outlier conditions, the average positioning error curves are generally below that of the Kalman and particle filter based methods, proving that our method has better robustness against respective outliers in both Wi-Fi fingerprinting and PDR positioning.

## 5. Conclusions

The Wi-Fi fingerprinting based positioning method has great potential for usage in indoor environments. To enhance the positioning accuracy, many types of assisted information can be adopted. The most commonly seen assisted information is the PDR information because it is normally available from a COTS smartphone. Filters such as Kalman filters and the particle filter are adopted in many methods for the integrated positioning. A new methodology is proposed in this paper which combines hierarchical Wi-Fi fingerprinting, Kalman filtering and graph optimization. According to the experiments: (1) the adoption of the hierarchical structure and the PLGD metric can improve the Wi-Fi based positioning accuracy; (2) with real-scenario data, our method was proven to be more accurate than classical Kalman filter and particle filter based methods (with less average positioning errors); (3) both real-scenario experiments and simulations have shown that the proposed method is more robust than the classical Kalman filter based method and particle the filter based method (with less maximum positioning error). Particularly, the proposed method performs better than classical Kalman filter and particle filter based methods if a lot of outliers exist in both Wi-Fi based fingerprinting and PDR results. The average position error is 1.7 and 2.0 m less than traditional Kalman filter based integration and particle filter based integration if 10% outliers are added in both Wi-Fi fingerprinting and PDR results.

## 6. Discussions and Future Work

The aim of this paper was to provide an approach for ubiquitous indoor positioning, especially for scenarios in public areas, such as malls, stations and airports. The proposed approach only requires a COTS smartphone and already-installed APs. In our approach, no extra hardware is needed and large-scale deployment costs are minimized. Therefore, it is suited for ubiquitous indoor positioning in a lot of public areas.

As mentioned, the complexity of the proposed optimization is $O(N^{2})$, where N is the number of poses to be estimated. Therefore, as the positioning time grows longer, the number of poses also grows, which can lead to significant latency in positioning. One simple strategy to alleviate the growing latency is to gradually discard error terms related to poses in early times, thereby giving the optimization problem an upper limit scale. However, in our experiment, the latency is not significant, since our longest trajectory only has 137 poses. The detailed strategy for lower the positioning latency will be studied in the future.

Other assistive techniques such as RFID, UWB, foot mounted positioning and so on are also very helpful for improving positioning accuracy. However, compared to Wi-Fi networks, normally they need to be installed on purpose, thereby increasing the system cost. Overall, the cost-efficiencies of other assistive techniques will be studied in the future.

Specially, if some satellite based results are available in the outdoor environment, they can be adopted in the proposed method. The CCF can then include terms derived from satellite based observations, and thus improve the overall accuracy. The adoption of satellite based information in the outdoor environment will also be studied in the future.

## Figures and Tables

**Table 1:** The average and maximum positioning error comparisons using different methods.

Method	Average Error (m)	Max Error (m)
pure Wi-Fi based	4.2	15.1
proposed method	2.9	8.4
Kalman filter	3.7	9.7
particle filter	3.6	9.4

**Table 2:** The average and maximum positioning error comparisons if 5% of outliers in Wi-Fi fingerprinting are added.

Method	Average Error (m)	Max Error (m)
proposed method	3.2	8.7
Kalman filter	4.3	10.7
particle filter	4.1	10.8

**Table 3:** The average and maximum positioning error comparisons if 10% of outliers in Wi-Fi fingerprinting are added.

Method	Average Error (m)	Max Error (m)
proposed method	3.5	9.2
Kalman filter	5.2	15.2
particle filter	5.4	14.9

**Table 4:** The average and maximum positioning error comparisons if 5% of outliers in PDR positioning are added.

Method	Average Error (m)	Max Error (m)
proposed method	3.7	8.5
Kalman filter	4.5	11.4
particle filter	4.5	11.9

**Table 5:** The average and maximum positioning error comparisons if 10% of outliers in PDR positioning are added.

Method	Average Error (m)	Max Error (m)
proposed method	4.2	11.2
Kalman filter	5.9	17.2
particle filter	5.7	16.9

**Table 6:** The numbers and their corresponding outlier conditions.

Numbers	Outlier Conditions
1	no outliers
2	5% Wi-Fi outlier
3	5% PDR outlier
4	5% Wi-Fi outlier + 5% PDR outlier
5	10% Wi-Fi outlier
6	10% PDR outlier
7	5% Wi-Fi outlier + 10% PDR outlier
8	10% Wi-Fi outlier + 5% PDR outlier
9	10% Wi-Fi outlier + 10% PDR outlier
